# LPG Cars in a Car Park Environment—How to Make It Safe

**DOI:** 10.3390/ijerph16061062

**Published:** 2019-03-24

**Authors:** Dorota Brzezińska

**Affiliations:** Department of Chemical Engineering, Lodz University of Technology, Faculty of Process and Environmental Engineering, Stefana Żeromskiego 116, 90-924 Lodz, Poland; dorota.brzezinska@p.lodz.pl

**Keywords:** LPG, accidental ventilation, CFD, explosive hazards

## Abstract

Alternative and innovative fuel types are being introduced to power cars. These include liquified petroleum gas (LPG) gas and hydrogen energy sources. However, they also introduce new hazards, requiring revised thinking with respect to safety within car parking environments. One of the most significant dangers is accidental gas release from a car’s system, especially in underground car parks. Jet fan systems are widely used for ventilation of such enclosures, but currently their design is most often based on computational fluid dynamics (CFD) according to computer simulations that may not be relevant for such new fuels. This paper presents the results of full-scale tests which demonstrate the operational factors of jet fan ventilation systems, and assesses the conditions which can occur in a car park when a small volume of LPG is released. On the basis of measurements undertaken, Fire Dynamics Simulator (FDS) software was validated against the air velocity flows and LPG gas dispersion patterns. Finally, the simulations were used to demonstrate the effectiveness of systems in an actual car park, in the case of an accidental LPG car tank release.

## 1. Introduction

Liquified petroleum gas (LPG) is gaining popularity around the world as a car fuel. Approximately 16 million vehicles are powered by this gas. It provides about 3% of the total fuel world market share. Almost half of all passenger vehicles fuelled by LPG gas can be found in the five largest markets: Turkey, Poland, South Korea, Italy and Australia. The biggest motivation for using LPG is the relatively low price, at approximately 40% of conventional fuels price. Another notable advantage is that LPG creates less pollutants than gasoline or oil. The problem is that, in reality, LPG car installations are very often in poor condition, creating the danger of accidental gas release [1,2,3,4,5,6,7].

**Figure 1:**
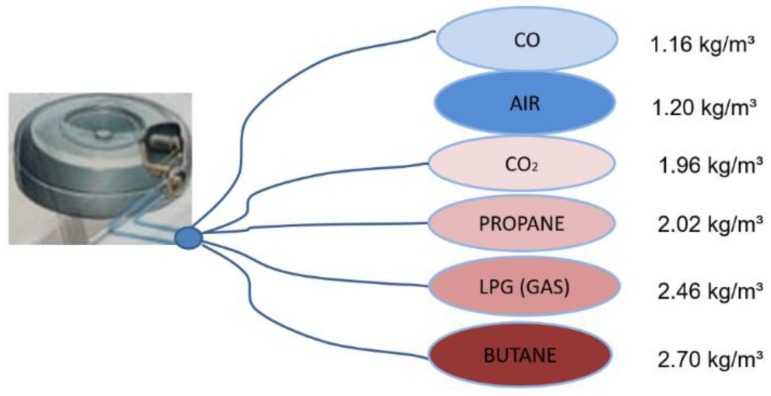
Liquified petroleum gas (LPG) gas density compared to other gases.

LPG is a mixture of propane (C_3_H_8_) and butane (C_4_H_10_). LPG mixtures contain a little more propane in winter and a little more butane during summer but, on average, the makeup is approximately 65% butane and 35% propane. The fluid phase LPG flowing out expands, cools, moves down and spreads along the ground and evaporates. One litre of liquid LPG produces 250 L of gas vapour. This means that from 0.17 × 10^−3^ m^3^ of liquid LPG (a typical volume of a car’s fuel system other than the tank) 42.5 × 10^−3^ m^3^ of pure gas can be created. After release, the pure gas thins in the air to 425 × 10^−3^ m^3^ of 10% volume condensation gas and later into 2.125 m^3^ of 2% volume condensation gas. At atmospheric pressure and normal temperature, LPG exists in a gas phase and its volume condensation of 10% corresponds to the upper explosive limit (UEL) and 2% to the lower explosive limit. The gas volume can be minimized in the high pressure when the gas can be liquefied. When the pressure decreases, LPG moves into the gas phase. This occurs during accidental gas release. A very important parameter is LPG density, with the gas being almost twice as heavy as air (Figure 1). LPG vapour can be transported along the car park floor over relatively long distances, reaching a source of ignition where the gas can ignite, flash back, or lead to serious vapour/air explosion hazards in confined spaces [8].

Despite the fact that LPG gas is so often used as a car fuel, the problem of its hazardous nature in enclosed car parks was evaluated only by computation field dynamics (CFD) analyses, without confirmation by experimental research into actual LPG scenarios [3]. However, there are some publications describing technical aspects of safe LPG transportation [9], refuelling at stations [10] or storage in tankers [11]. There are hardly any reports and guides related to relatively small LPG tanks and systems, such as those used in cars, when kept in relatively small enclosures such as car parks [9]. The article by Van den Schoor analyses theoretical scenarios of LPG gas leakage in a car park, but his conclusions are based only on CFD simulations [3]. In the literature there is a lack of data covering the risks of LPG installation/system failure in garages. Likewise, no standards exist for the effective detection and ventilation of LPG in such enclosures. Many countries address that problem by prohibiting the entrance of LPG-fuelled cars into closed car parks, but this is inconvenient for their users and very often is not respected [12].

The risk level in the case of flammable gas release depends on the gas flammability and its dispersion abilities in confined spaces [13,14]. Because of the lack of any statistics on the accidental failure of LPG car installations, the important data in this field is based on surveys from vehicle technical control stations, where each car has to be tested at least once a year. Such data shows that unsealed tanks are not registered but the gas leaks occur very often in the installation, especially at the pipe joints. Despite this, complete tank leakage cannot be excluded from consideration. LPG release from a car installation (as well as hydrogen in the case of such powered cars) can be detected and the risk of an explosion can be mitigated by automatically activated ventilation, especially with a jet fan system [12,15].

Jet fans which are used for ductless stream ventilation systems are often applied for ventilation in enclosed car parks [16]. The jet fans are installed directly under the ceiling of the garage and create an air flow which transports and removes the pollutants or dangerous gases [17]. Originally, jet fan systems were used only for the ventilation of tunnels, but recently they have become very popular for car parks [18,19]. Because of the relatively high density of the gas from LPG, the most important factor for its removal is having highly effective ventilation in place for the space near the floor of the car park. Consequently, this requires consideration of the design of such systems [12].

Compared with duct ventilation systems, CFD modelling of jet fans ventilation creates more difficulties. The reason is due to the high speed and dynamics of the air flowing out from the fan. In addition, at the fan’s outlets, guide vanes are usually installed, which change the air stream direction towards the floor. Effective CFD modelling of jet fan systems requires detailed validation works and proper turbulence coefficients for resolving the Navier–Stokes equations [12,20,21].

For the CFD analyses presented in this article, the software Fire Dynamics Simulator (FDS 6) was used, which solves the Navier–Stokes equations appropriate for the fluid flows appearing in natural and mechanical ventilation, sprinklers, nozzles outflows, etc. [20,21].

**Figure 2:**
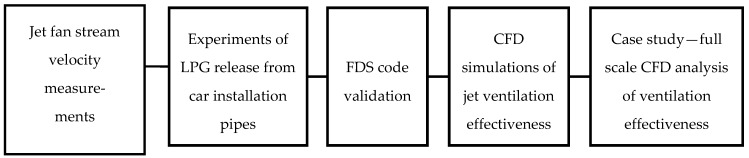
Steps in the analysis process. FDS: Fire Dynamics Simulator; CFD: computational fluid dynamics.

The main goal of the experimentation and analysis was to validate the FDS model as a tool that can be used to predict the dispersion of gas from the LPG car tank and the efficiency of jet fan air streams. To do so, this study made use of the comparison of full-scale experiments with computer modelling. For safety reasons, full-scale tests using full tank release could not be completed and these scenarios were analysed only using the FDS code. Figure 2 presents subsequent steps of the analysis.

Table 1 presents a synthesis of unsolved problems mentioned in the literature related to LPG-fuelled cars parked in underground car parks, which were the subject of research described in the article.

## 2. Real-Scale Tests and CFD Simulations

### 2.1. The Jet Fan Stream Velocity and Its Influence on the Gas Clouds

The first stage of experiments involved the jet fan stream velocity measurements and its CFD modelling, which are described below.

#### 2.1.1. Measurement Layout for Jet Fan Stream Velocity

**Figure 3:**
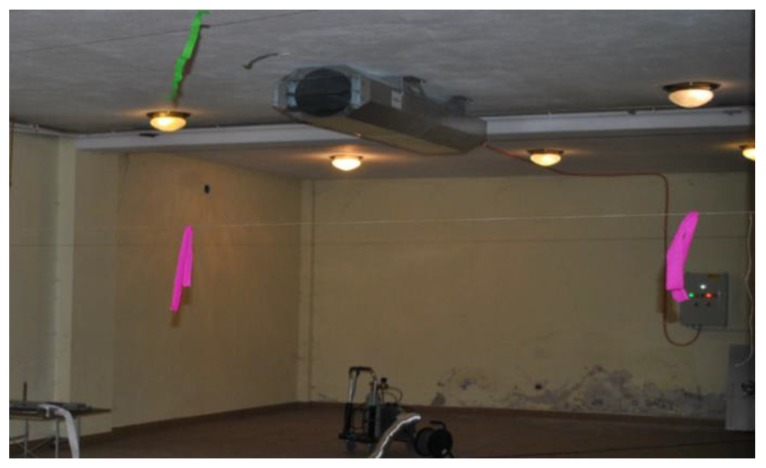
Photo of the test room.

**Figure 4:**
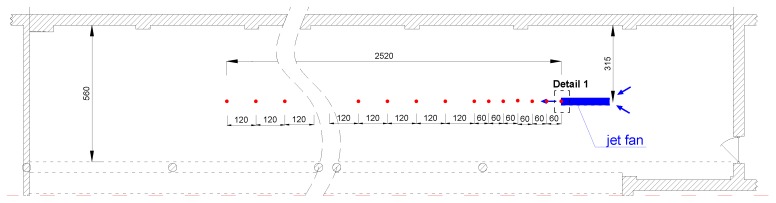
Test room scheme with measuring points presentation.

For the experiments, a typical jet fan of 315 × 10^−3^ m diameter and 1.18 m^3^/s volume flow was used (FläktWoods Sp. z o.o., Ołtarzew, Poland, model 31JT-3LP-UBD-TB). The tests were carried out in a large enclosure, on the premises of the Lodz University of Technology. The research was conducted on a test bench of plan dimensions 40 m × 4.8 m and height of 3.1 m with the wall on the one side of the measuring space, and pillars on the other. The analysis used measurements made in the axis of the jet fan at a distance of 25.2 m from its outflow. Distances between the measuring points along the airstream were 0.6 m apart when close to the ventilator, whereas they were 1.2 m apart when further away. Speed measurements were made at heights of 0.3 m, 1.5 m and 2.8 m from the floor. A photo of the test room is presented in Figure 3 and a plan illustrating the measuring points is shown in Figure 4.

The tests of the jet fan air speed were carried out using the jet fan with guide vanes sloped towards the floor, at an angle of 30° to the longitudinal axis of the fan, as is usually used in actual systems. The air speed in the airstream was measured with a set of three sensors TESTO 0635.1049 connected to the logger TESTO 454, manufactured by Testo Sp. z o.o., Pruszków, Poland. The velocity range of the sensors was 0–10 m/s with an accuracy < 0.01 m/s ± 5% of the measured value. Measurements recorded by the meter were developed using the Comsoft3 TESTO computer software. The air speed was measured at each measurement point every 1 s for 10 s. The final air speed was taken as the arithmetic average of 10 measurements.

#### 2.1.2. Measurement Results of Jet Fan Stream Velocity

**Figure 5:**
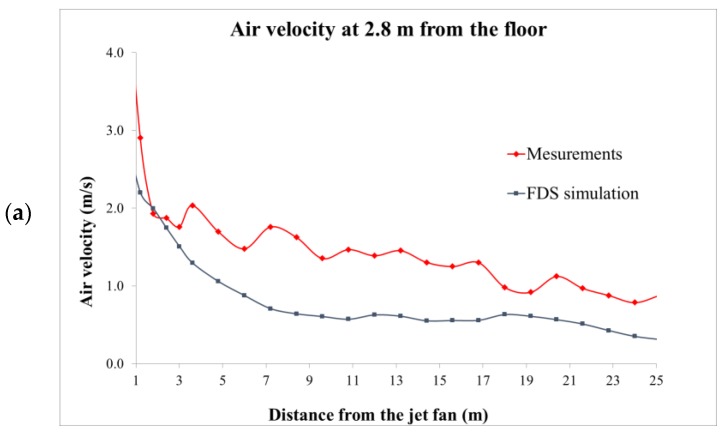

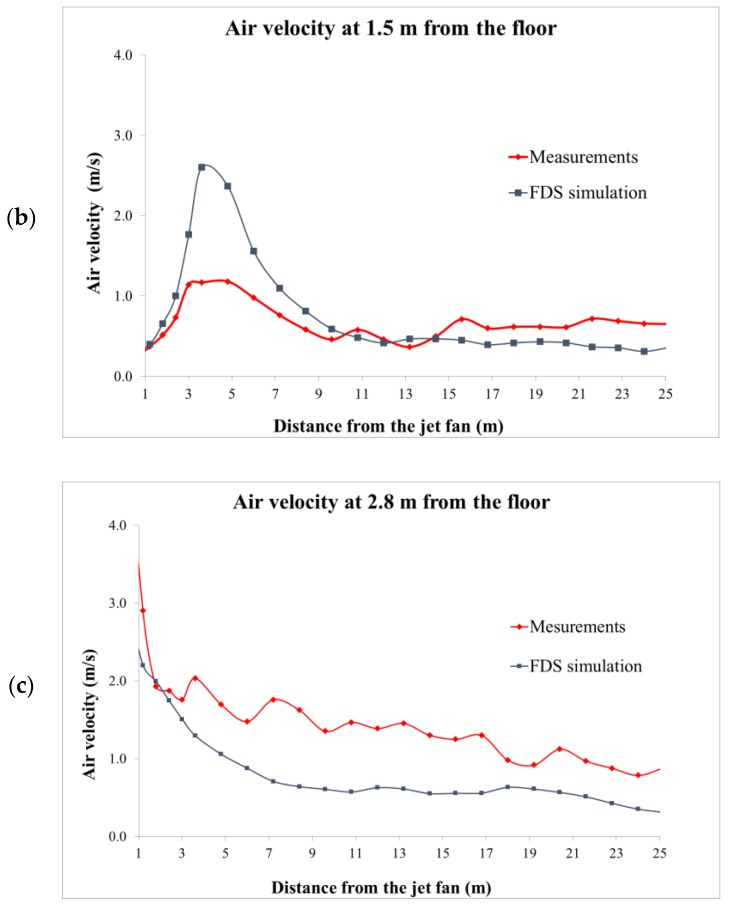
Results of the jet fan airflow velocity measurements and FDS simulations at heights from the floor of (**a**) 0.3 m; (**b**) 1.5 m; and (**c**) 2.8 m.

Figure 5 shows the results of the measurements of the jet fan airflow speed. The curves represent axis air velocity at 0.3 m, 1.5 m and 2.8 m from the floor, however, for the removal of LPG the height of 0.3 m is the most significant. Looking at the chart in Figure 5a, it can be concluded that air speeds at 0.3 m appear at a distance of 4 m from the fan and keeps the value between 0.3 m/s and 0.6 m/s at the distance above 25 m.

#### 2.1.3. FDS Code Validation for Jet Fan Stream Velocity

For the CFD simulations, a 3D model of the measurement enclosure was prepared. FDS version 6.1.1 was used for the simulations. The grid density in the simulation used was 0.15 m and the turbulence was modelled with Deardorff’s model used for the turbulence viscosity representation [20]. In accordance to the considerations regarding jet fans that were described in the introduction, the input parameters for the jet fan in FDS software were as follows:

&OBST XB = 3.4,5.1,3.75,4.05,2.55,2.85, SURF_ID = ‘INERT’/
&VENT ID = ‘Vent11’, SURF_ID = ‘HVAC’, XB = 3.4,3.4,3.75,4.05,2.55,2.85, IOR = −1, RGB = 255,51,51/
&VENT ID = ‘Vent12’, SURF_ID = ‘HVAC’, XB = 5.1,5.1,3.75,4.05,2.55,2.85, IOR = 1, RGB = 255,51,51, UVW = 1.0,0, −0.308/
&HVAC ID= ‘Duct05’, TYPE_ID = ‘DUCT’, DIAMETER = 0.15, FAN_ID = ‘Fan01’, NODE_ID = ‘Node11’,’Node12’, ROUGHNESS = 0.001, LENGTH = 1.7/
&HVAC ID = ‘Node12’, TYPE_ID = ‘NODE’, DUCT_ID = ‘Duct05’, VENT_ID = ‘Vent12’/
&HVAC ID = ‘Node11’, TYPE_ID = ‘NODE’, DUCT_ID = ‘Duct05’, VENT_ID = ‘Vent11’/
&HVAC ID = ‘Fan01’, TYPE_ID = ‘FAN’, VOLUME_FLOW = 1.18, DEVC_ID = ‘TIMER’/

Figure 5 presents the simulated air velocities against the measurements, at the heights of 0.3 m, 1.5 m and 2.8 m from the floor.

The simulation results were closely comparable with measured air velocities at all verified heights from the floor. The tendency of the air velocity to decrease at increasing distances from the jet fan was correctly mapped, however, at the height of 2.8 m the simulations overestimated this parameter, which should be taken into account in real ventilation system projects.

**Figure 6:**
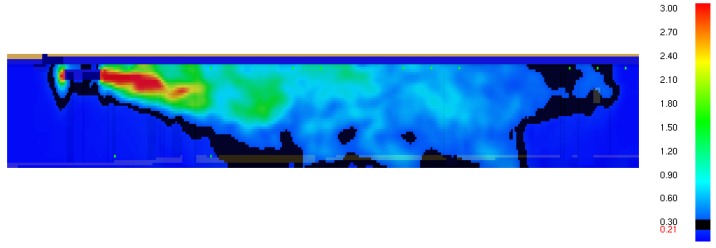
Graphical presentation of a simulated air stream velocity. The scale presents velocity range in (m/s).

Figure 6 presents a simulated airstream velocity. It is shown that the air speed distribution in the vertical plane which passes through the axis of the jet fan confirms experimental results, and the air velocity near the floor appears at a distance of about 4 m from the jet fan. The black colour in the picture represents a speed of 0.2 m/s, which is treated as the minimum speed needed to remove air pollution from a garage.

#### 2.1.4. Jet Fan System Effectiveness

**Figure 7:**
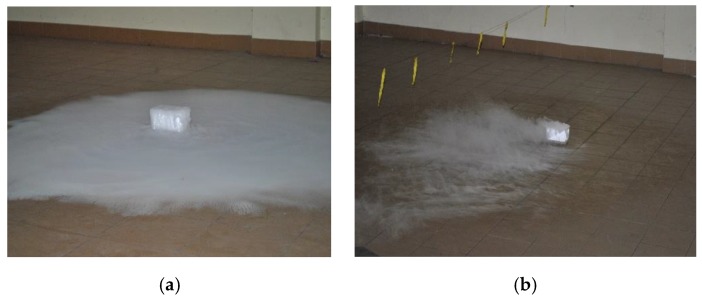
The dry ice test results: (**a**) jet fan ventilation system turned off; (**b**) jet fan ventilation system activated.

During the first approach of the experiment evaluating ventilation effectiveness, LPG was replaced by dry ice, which generated a CO_2_ cloud, which has similar parameters of density and dispersion as LPG. The test functioned as a qualitative evaluation of the jet fan ventilation system. It was observed that without ventilation gas cumulated at the floor, but when the jet fan ventilation worked, the gas was removed immediately (this is presented in Figure 7).

### 2.2. The LPG Dispersion Experiments

The second stage of experimentation used LPG gas released from the car installation. During the tests, gas dispersion measurements as well as CFD simulations were conducted. The results are described below.

#### 2.2.1. Measurement Layout for LPG Dispersion

**Figure 8:**
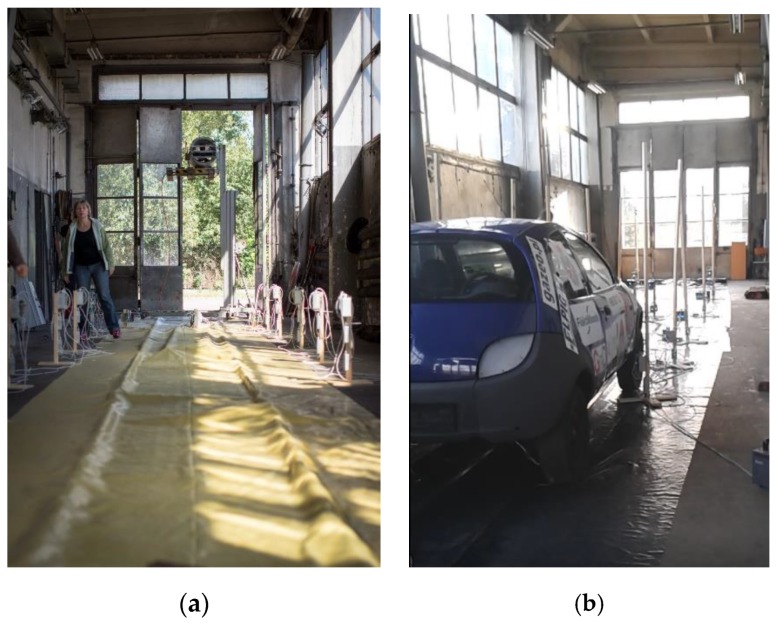
Photos of the measurement layout for the LPG experiments: (**a**) the full enclosure view, (**b**) the car body and measurement instruments.

**Figure 9:**
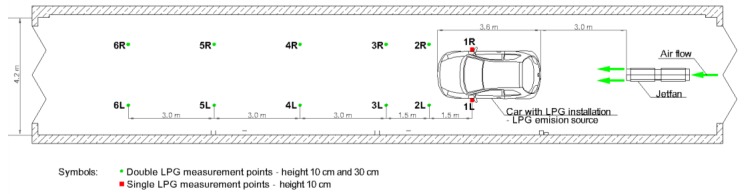
Plan for the spacing of the measurements during the LPG dispersion experiment.

The LPG gas release measurements were realised in an enclosure of dimensions 23.7 m × 4.2 m, and a height of 6 m (shown in Figure 8 and Figure 9, respectively). The LPG installation was installed in a real car body, and gas concentration was measured with the semiconductor-type gas sensors TGS2610 manufactured by Figaro Engineering Inc., Osaka, Japan. These are semiconductor-type gas sensors which have a very high sensitivity to LPG gas. The optimal detection concentration of TGS2610 detectors ranges between 500 ppm and 10,000 ppm of iso-butane and propane. The experiment assumed the outflow of the full volume of the pipe dedicated for fluid phase LPG which was of 0.17 × 10^−3^ m^3^, as described in the introduction.

The same jet fan that was used in the first stage of the study was used for the LPG experiments. The fan was installed in the measurement enclosure at the height of 2.5 m from the floor. During the tests, the jet fan’s guide vanes were sloped towards the floor in order to create adequate air flow speed near the floor.

**Figure 10:**
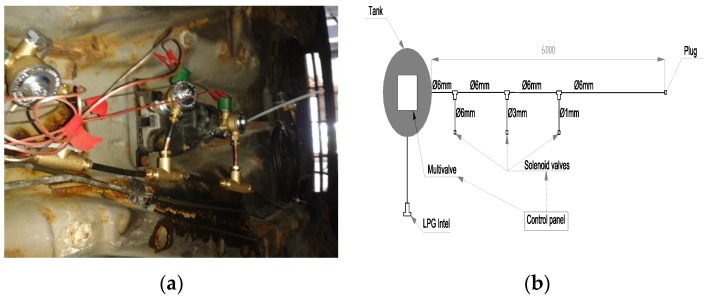
Photo (**a**) and scheme (**b**) of the car installation used during the LPG dispersion experiment.

For the experiments, a typical car installation for LPG was used, which included all the elements contained in the standard LPG installation, in its liquid phase section. Figure 10 shows the scheme and photo of the installation. Three remote solenoid valves were installed in the LPG transport valve for imitation of gaps in the pipe. Each valve had an outlet with a different diameter—1 mm, 3 mm, and 6 mm, respectively.

Six tests were conducted to investigate the gas outflow phenomenon from the car installation and its dispersion in the enclosure. The first three tests were realized with the jet fan turned off. For each available diameter of opening (1 mm, 3 mm, 6 mm) gas condensation at the measurement points shown in Figure 8 was registered. All the probes were also repeated with the ventilation system activated. The jet fan switched on automatically when the detector located at a distance of 9 m from the gas source found the gas condensation to be equal to 10% of the LPG lower explosion limit.

#### 2.2.2. Measurements of LPG Dispersion Results

**Figure 11:**
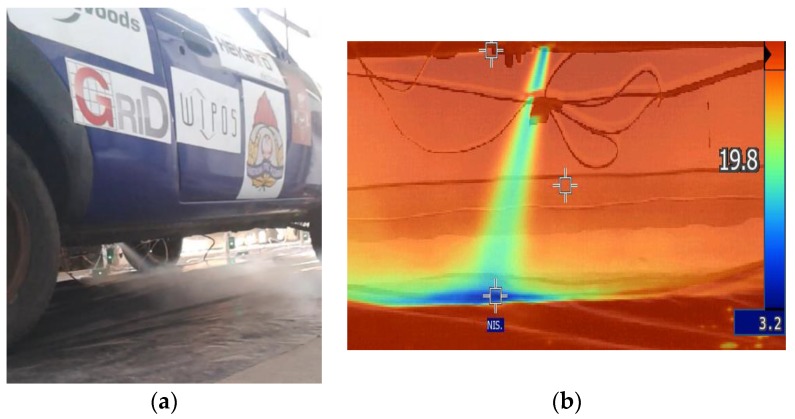
Photos of the LPG emission phenomena: (**a**) LPG gas cloud under the car; (**b**) LPG stream temperature range (an infrared camera photo).

The experiments show that, in a case of LPG car installation failure, the gas escapes, evaporates and disperses (Figure 11a). The transition of LPG from liquid phase into gas is a very strong endothermic process, as was confirmed by using an infrared camera (Figure 11b), which shows a cold LPG gas stream flowing out from the car installation.

**Figure 12:**
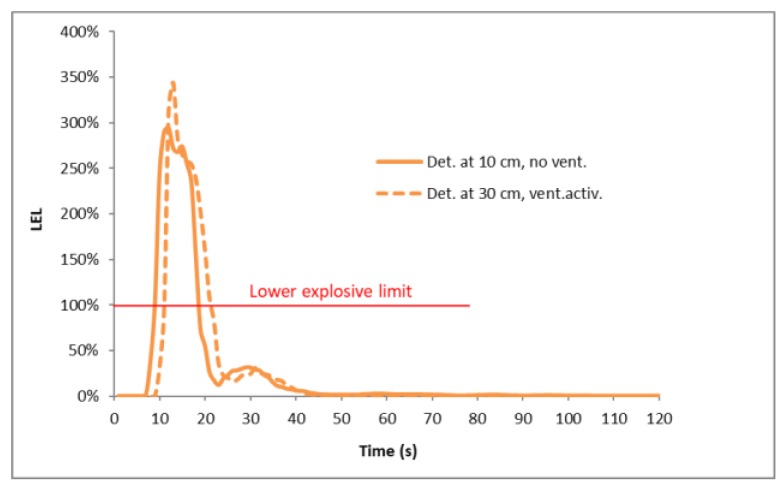
LPG concentration close to the emission source for 6-mm gap (measurement point No. 1R).

**Figure 13:**
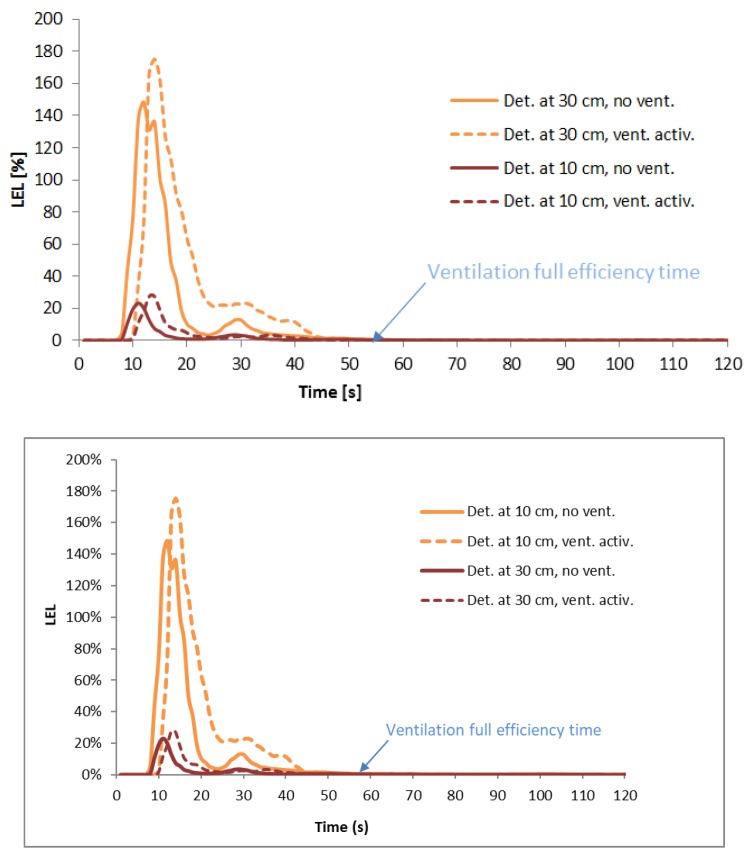
LPG concentration 3 m from emission source with 6-mm gap (measurement point No. 3L).

The highest LPG gas concentration was observed at a very short distance from the emission source (measurement point No. 1R in Figure 9) and the maximal values were reached during the probe with the largest gap diameter (6 mm), when the condensation reached 350% of the lower explosive limit. The high concentration (representing the possibility of an explosion) appeared for about 10 s and later decreased on its own (Figure 12). At greater distances from the emission source (measurement point No. 3L in Figure 9), the LPG concentration reached much lower levels. Furthermore, a huge difference in gas concentration between detectors located at heights of 0.10 m and 0.30 m was observed. This proved that there was a much higher probability of LPG detection very close to the floor than at the height of 30 cm which is the standard height for detectors (Figure 13).

#### 2.2.3. FDS Code Validation for LPG Dispersion

**Figure 14:**
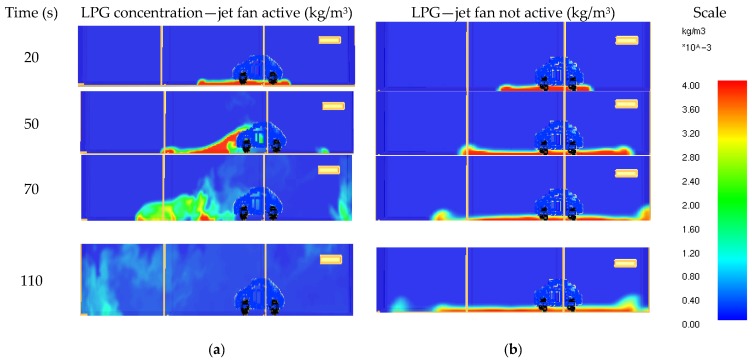
CFD results—LPG gas concentration: (**a**) jet ventilation active and (**b**) jet ventilation not active.

On the basis of the prescribed measurements, a 3D model of the measured space and gas dispersion simulations was prepared. FDS Version 6.1.1 was used for the simulations, and the turbulence was modelled with Deardorff’s model used for the turbulence viscosity representation [17]. Figure 14 shows the LPG concentration in the situation results for when the jet fan was turned on and off. The simulations confirmed the significant role of ventilation system on the LPG dispersion phenomena. For the scale in Figure 14, the condensation of 4·10^−3^ kg/m^3^ a 10% lower explosive limit (LEL).

**Figure 15:**
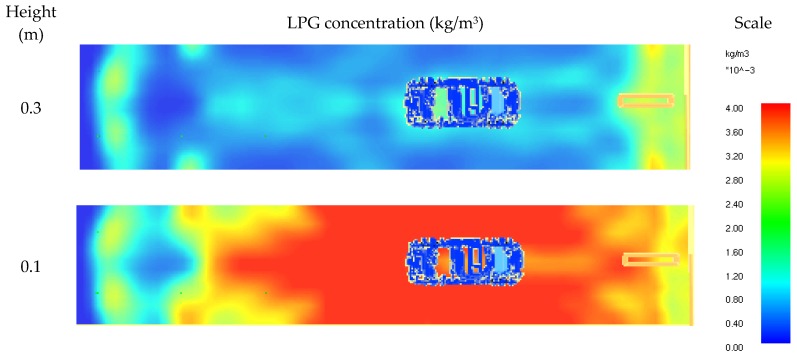
CFD results—LPG gas concentration at the height of 0.3 m and 0.1 m.

The simulations also confirmed that the concentration of LPG at the height of 0.3 m (the standard height used for detection) is several times lower than at the level of 0.1 m (see Figure 15).

## 3. Analysis of Ventilation Effectiveness on LPG Removal in a Full-Scale Car Park

**Figure 16:**
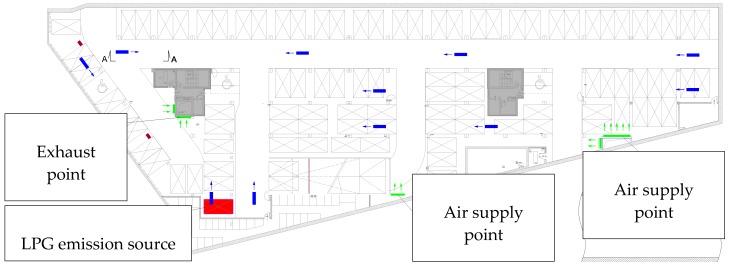
The ventilation system scheme in a real car park.

On the basis of the experiments and simulations described above, it was assumed that FDS is a computer software program capable of analysing the jet fan ventilation systems and performing a safety evaluation in a full-scale car park in the case of a car LPG system failure. The simulations were prepared for an actual garage with an area of 2900 m^2^. The total ventilation volume was taken as 24,000 m^3^/h (the volume appointed on the basis of the standard capacity for daily ventilation). The ventilation utilised one exhaust point, two points of fresh air supply and 11 jet fans. The plan of the car park is shown in Figure 16.

**Figure 17:**
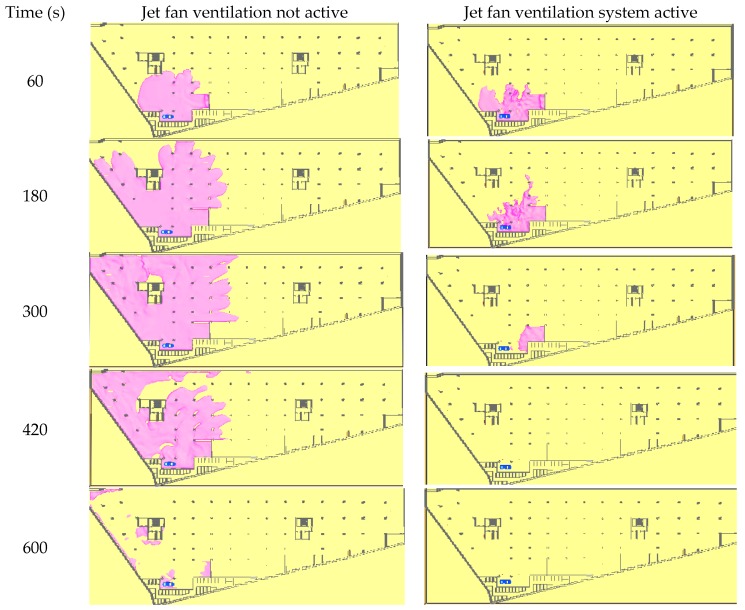
The range of the LPG flammable gas cloud in the seconds following the gas release.

All assumptions and models taken for this exemplar simulation, like the jet fan stream, LPG release and dispersion models were based on FDS code validation results (described in Section 2 of this article). For the analysis, a continuous release of the full tank of 74 × 10^−3^ m^3^ liquid LPG was assumed. Two simulations were conducted. The first one assumed that only the exhaust and supply points of the ventilation system were working and the second one assumed that additionally 11 jet fans were activated and operated in the direction shown in Figure 16. The results for the two cases were compared and are presented in Figure 17. The pink colour cloud in the pictures represents the volume where the LPG concentration exceeded the lower explosive limit. The simulation results are presented in the seconds following the LPG release. The simulation results confirmed that the jet fans were able to thin the LPG gas concentration quickly, and only a period of five minutes was required for elimination of the explosion risk in the car park.

## 4. Conclusions

Experimentation was undertaken of jet fan air stream velocities with accidental LPG dispersion in a car park. The CFD simulations based on the same conditions as those used during the experiments confirmed that FDS software can be used for the design of jet fan ventilation systems as well as LPG release and dispersion in the car park. Based on the experimental and CFD simulations results, the following observations and conclusions were formulated:

1. Following accidental LPG release, without suitable ventilation systems, the gas accumulates on the floor of the car park, creating a significant explosive hazard;
2. LPG detection should be located as close as possible to the floor, as even at a height of 0.30 m from the floor the detectors may not be effective;
3. properly designed ventilation (especially jet fan systems) can efficiently remove LPG from the car park, even in the case of a huge gas leakage from a car tank.

These practical recommendations for car park designers and stakeholders should not require excessive additional costs in car parks where smoke control systems are required by local regulations, because the same systems could be used for the LPG control. In the case of car parks without such requirements, the additional costs of LPG control systems could be significant. In such cases, decisions may need to be made regarding whether or not LPG-fuelled cars are allowed into the car park.

## Figures and Tables

**Table 1:** Problems related with LPG fuelled cars parked in underground car parks.

The Problem	The Realized Research
The jet fans airstream velocities are not fully known and modelling with FDS software has not been validated	The jet fans airstream velocities were measured and the experimental results were compared with FDS simulations (Section 2.1)
The LPG dispersion process in the enclosures is not fully known and its modelling with FDS software has not been validated	LPG concentrations in a case of low volume gas release from a car tank were measured in real-scale experiments and the results were compared with FDS simulations (Section 2.2)
The effectiveness of jet fan ventilation systems on LPG explosive cloud size in a case of full car tank accidental release has not been confirmed	Analyses of the effectiveness of jet fan ventilation systems on removing LPG in the real-scale car park were conducted on the basis of FDS simulations (Section 3)

## Abbreviations

CFD	Computational Fluid Dynamics
FDS	Fire Dynamics Simulator
LEL	Lower Explosive Limit
LPG	Liquefied Petroleum Gas
UEL	Upper Explosive Limit
